# Single-cell characterization of macrophages in uveal melanoma uncovers transcriptionally heterogeneous subsets conferring poor prognosis and aggressive behavior

**DOI:** 10.1038/s12276-023-01115-9

**Published:** 2023-11-01

**Authors:** Ke Li, Lanfang Sun, Yanan Wang, Yixin Cen, Jingting Zhao, Qianling Liao, Wencan Wu, Jie Sun, Meng Zhou

**Affiliations:** 1 State Key Laboratory of Ophthalmology, Optometry and Visual Science, Eye Hospital, Wenzhou Medical University, 325027 Wenzhou, China; 2 National Clinical Research Center for Ocular Diseases, Eye Hospital, Wenzhou Medical University, 325027 Wenzhou, China

**Keywords:** Eye cancer, Prognostic markers, Predictive medicine

## Abstract

Uveal melanoma (UM) is the most frequent primary intraocular malignancy with high metastatic potential and poor prognosis. Macrophages represent one of the most abundant infiltrating immune cells with diverse functions in cancers. However, the cellular heterogeneity and functional diversity of macrophages in UM remain largely unexplored. In this study, we analyzed 63,264 single-cell transcriptomes from 11 UM patients and identified four transcriptionally distinct macrophage subsets (termed MΦ-C1 to MΦ-C4). Among them, we found that MΦ-C4 exhibited relatively low expression of both M1 and M2 signature genes, loss of inflammatory pathways and antigen presentation, instead demonstrating enhanced signaling for proliferation, mitochondrial functions and metabolism. We quantified the infiltration abundance of MΦ-C4 from single-cell and bulk transcriptomes across five cohorts and found that increased MΦ-C4 infiltration was relevant to aggressive behaviors and may serve as an independent prognostic indicator for poor outcomes. We propose a novel subtyping scheme based on macrophages by integrating the transcriptional signatures of MΦ-C4 and machine learning to stratify patients into MΦ-C4-enriched or MΦ-C4-depleted subtypes. These two subtypes showed significantly different clinical outcomes and were validated through bulk RNA sequencing and immunofluorescence assays in both public multicenter cohorts and our in-house cohort. Following further translational investigation, our findings highlight a potential therapeutic strategy of targeting macrophage subsets to control metastatic disease and consistently improve the outcome of patients with UM.

## Targeting Macrophage Subsets: A Potential UM Therapy

A recent study utilized single-cell transcriptome data to explore the cellular heterogeneity of macrophages within the uveal melanoma (UM) tumor microenvironment, revealing four distinct macrophage subsets. One subset, MΦ-C4, was found to be associated with aggressive tumor behavior and poor survival outcomes. The study also developed a machine-learning-based subtyping system to classify UM subtypes and predict prognosis based on MΦ-C4-specific core metagenes. This research deepens our understanding of cellular heterogeneity in UM and highlights the potential therapeutic benefits of targeting macrophages in UM treatment.

## Introduction

Uveal melanoma (UM) arises from melanocytes in the uveal tract and is the most common primary intraocular malignancy among adults, accounting for more than 85% of all ocular melanomas^1,2^. Local treatments such as conservative irradiation therapies and surgical excisions have been widely used to control primary UM, improve patient prognosis, and enhance survival rates and quality of life^3,4^. However, UM is a highly metastatic disease, with ~50% of patients developing distant metastases that result in fatal outcomes, with a median overall survival (OS) of 12 months^5–7^. Hence, there is an urgent need for improved therapeutic options and targets to effectively treat UM metastases and improve long-term survival rates.

Research on UM genetics and pathogenesis has revealed the heterogeneous nature of this disease, leading to the identification of molecularly and clinically distinct subtypes^2,8–13^. Tumors are complex ecological systems composed of cancer cells, nonmalignant stromal cells, and immune cells, collectively known as the tumor microenvironment (TME)^14,15^. Although the eye is a critical immunologically privileged site, evidence has shown that the immunological heterogeneity in the TME markedly impacts various aspects of UM biology, including development, progression, metastasis, prognosis, and treatment response^16–21^. Prior studies have characterized the immune infiltration involved in UM from bulk tissue data and identified monocytes or macrophages of the M2 lineage as the predominant group of tumor-infiltrating immune cells; these cells are associated with tumor growth and poor prognosis^16,22,23^. Recent advances in single-cell RNA sequencing have highlighted the heterogeneous nature and functional diversity of macrophages, independent of the M1/M2 model^24^. For example, Chen et al. identified a novel macrophage subpopulation as a mesenchymal pro-tumor marker in gliomas^25^. A time series analysis of scRNA-seq during the acute phase of kidney injury confirmed the relevance of S100A8/A9+ macrophage infiltration in tissue injury^26^. A recent pancancer single-cell analysis of macrophage subsets also revealed a high level of complexity and heterogeneity in different cancer types, emphasizing the need to study the complex phenotype of macrophages in the TME separately^27^. However, the cell heterogeneity and functional diversity of macrophages have yet to be fully elucidated at the single-cell level in UM.

In this study, we conducted a single-cell RNA sequencing analysis of macrophages in patients with UM to characterize macrophage heterogeneity and identified four macrophage subsets. Then, we explored the clinical effects of four macrophage subsets on tumor phenotypes and prognosis using multicenter bulk and single-cell cohorts and evaluated their contribution to the molecular subtyping validated by using our in-house cohort by RNA-seq and immunofluorescence imaging. Our study provides important insights into the diversity of macrophages in UM and demonstrates the potential of targeting macrophage subsets to improve routine clinical practice.

## Materials and methods

### Single-cell RNA-seq data and analysis

scRNA-seq data (10x Genomics) and clinicopathological information of 11 patients with UM were retrospectively collected from Durante’s study in the Gene Expression Omnibus (GEO) database, under accession number GSE139829^28^.

scRNA-seq data processing and analysis were implemented using the Seurat v4 R package^29^. All functions were executed with default parameters unless otherwise stated. scRNA-seq data for all UM samples were first integrated using the merge function, and low-quality cells (<200 genes/cell or >8000 genes/cell and >10% mitochondrial genes) were removed. Following quality control, the scRNA-seq data were normalized using the SCTransform method with regression to determine the mitochondrial percentage^30^. The potential batch effect between samples was corrected using the Harmony method^31^. Principal component analysis was performed to reduce dimensionality, and the top 20 principal components were adopted to identify distinct groups of cells using the graph-based clustering method with the FindClusters function (resolution = 2). Major cell clusters were annotated using known cell-type marker genes and visualized using a T-distributed stochastic neighborhood embedding (tSNE) scatter plot. Differentially expressed genes (DEGs) between each cell type and all other cell types were identified using the FindAllMarker function in Seurat, and significance was determined using the Wilcoxon rank sum test with Bonferroni correction. Genes were selected as DEGs based on the threshold of |logFC| > 0.5 and an adjusted *P* value < 0.05.

### Bulk transcriptome data and analysis

Bulk RNA-seq data and clinicopathological information of 80 UM samples were obtained from UCSC Xena (GDC The Cancer Genome Atlas (TCGA)-UVM cohort; https://xenabrowser.net/datapages/). Gene expression levels measured using the HiSeq Illumina platform were quantified as FPKM-UQ and normalized using log2-transformation.

Bulk microarray data and clinicopathological information for 120 patients with UM were obtained from the GEO, including 63 patients from Laurent’s study (accession no. GSE22138)^32^, 29 patients from Gangemi’s study (accession no. GSE27831)^33^ and 28 patients from van Essen’s study (accession no. GSE84976)^34^.

The raw microarray data from the Human Genome U133 Plus 2.0 array were preprocessed and normalized using the robust multichip average (RMA) algorithm for background correction, quantile normalization, and log2 transformation. Processed expression data from the Illumina HumanHT-12 V4.0 expression beadchip provided by the authors were used^33^.

### Definition of signature scores

TEX score was defined by the mean expression value of TEX-related gene sets (PDCD1, CTLA4, LAG3, TIGIT, TOX, and TCF7). M1- and M2-MΦ signature enrichment scores were calculated with canonical M1- and M2-MΦ signatures using the “AddModuleScore” function in Seurat (Supplementary Table 1).

### Trajectory analysis

Dramatic translational relationships among cell types and clusters were determined using Monocle2 (v2.16.0) and CytoTRACE (https://cytotrace.stanford.edu/)^35,36^. For Monocle2, the CellDataSet object was created using the Cell DataSet function with the parameter “expressionFamily = negbinomial”. The differentiation trajectory was built using default parameters after dimensionality reduction. Genes for trajectory inference were determined using the dispersionTable function. Only genes with a mean expression > 1 were selected for analysis. Dynamic differentially expressed genes (DEGs) across pseudotimes were identified using the differentialGeneTest function with *q* < 0.001. For CytoTRACE, we input the expression count matrix of macrophages into the online tool and then obtained the result.

### SCENIC analysis

SCENIC analysis was employed to investigate transcription factor (TF) regulation with default parameters^37^. The expression count matrix from the Seurat object was filtered for genes using the geneFiltering function in Seurat with the default threshold (minCountsPerGene, 0.03 × ncells; minSamples, 0.01 × ncells) and normalized to the input. GRNboost was used to infer TF target gene coexpression modules, and RcisTarget was utilized to identify regulons with the default parameters and the following cisTarget databases: hg19-500 bp-upstream-7species.mc9nr.feather and hg19-tss-centered-10 kb-7species.mc9nr.feather. The activity of each regulon of single cells was scored using the AUCell method.

### Enrichment analysis

The enrichment score for a specific gene set or pathway activity was calculated using single-sample gene set enrichment analysis (ssGSEA) with the R package “GSVA”^38^. To conduct functional enrichment analysis of macrophage subsets, we identified sorted differentially expressed genes (DEGs) for each macrophage subset and performed gene set enrichment analysis (GSEA) using cancer hallmark pathways with the clusterProfiler (v3.18.0) package^39^.

### Patient biospecimen collection and RNA extraction

The present study was performed according to the Declaration of Helsinki and approved by the Ethics Committee of the Eye Hospital of Wenzhou Medical University (ethics approval no. 2022-043-K-28-02). All patients provided written informed consent. All data were anonymously analyzed. Human UM tissues were collected during surgical resection at the Eye Hospital of Wenzhou Medical University from five patients diagnosed with UM. The patients did not receive preoperative treatment prior to collection.

Total RNA from UM tissues was isolated using TRIzol^®^ Reagent (Invitrogen, Thermo Fisher Scientific, Inc.). The OD_260_/OD_280_ ratio was used as an indicator of RNA purity; the ratio is required to be close to 2.0 for pure RNA (acceptable range, 1.8–2.1). RNA concentration and purity were measured using a NanoDrop 2000 Spectrophotometer (Invitrogen, Thermo Fisher Scientific, Inc.).

### Immunofluorescence assay

Tumor tissues were fixed in 4% paraformaldehyde (PFA) at 4 °C overnight, dehydrated in 10%, 20%, and 30% sucrose solution, transferred into OCT, and frozen at −80 °C for subsequent use. Tissues with a thickness of 10 μm were prepared and washed using phosphate-buffered saline (PBS). Primary antibodies against CD68 (dilution 1:200; cat. no. sc-20060, Santa Cruz Biotechnology, Inc.) and Ferritin Light Chain (dilution 1:100; cat no. ab69090; Abcam) were applied. Tissue sections were incubated with primary antibodies at 4 °C overnight and then with secondary antibodies, Alexa Fluor 594-labeled donkey anti-rabbit IgG [(H + L); cat no. 34212ES60; Shanghai Yeasen Biotechnology Co., Ltd.] and Alexa Fluor 488-labeled donkey anti-mouse IgG [(H + L); cat. no. 34106ES60; Shanghai Yeasen Biotechnology Co., Ltd.). Nuclei were counterstained with DAPI, and signals were visualized using a DM4B (Leica Microsystems GmbH).

### Statistical analysis

All statistical analyses were conducted in R (v4.0.3) with the R studio interface (v1.3.959). Wilcoxon rank-sum or Student’s *t*-tests were used to compare differences between the two groups. Consensus clustering analysis was conducted using the R package “ConsensusClusterPlus” with the following parameters: pItem = 0.8, pFeature = 1, reps = 1000, and the “Pam” clustering approach. Univariate and multivariate Cox proportional hazard regression models were utilized to assess the association between variables and survival time. The Kaplan‒Meier method and log-rank test were used to compare survival differences between different patient groups. Hazard ratios (HRs), 95% confidence intervals (CIs), and corresponding *P* values were calculated, and visualization of covariate effects was carried out using the forest plot with the R package “forestplot”. Spearman’s rank correlation coefficient was applied for correlation analysis. Statistical significance was set at *P* < 0.05.

## Results

### Single-cell transcriptomic analysis revealed four transcriptionally distinct macrophage subsets in UM

**Fig. 1:**
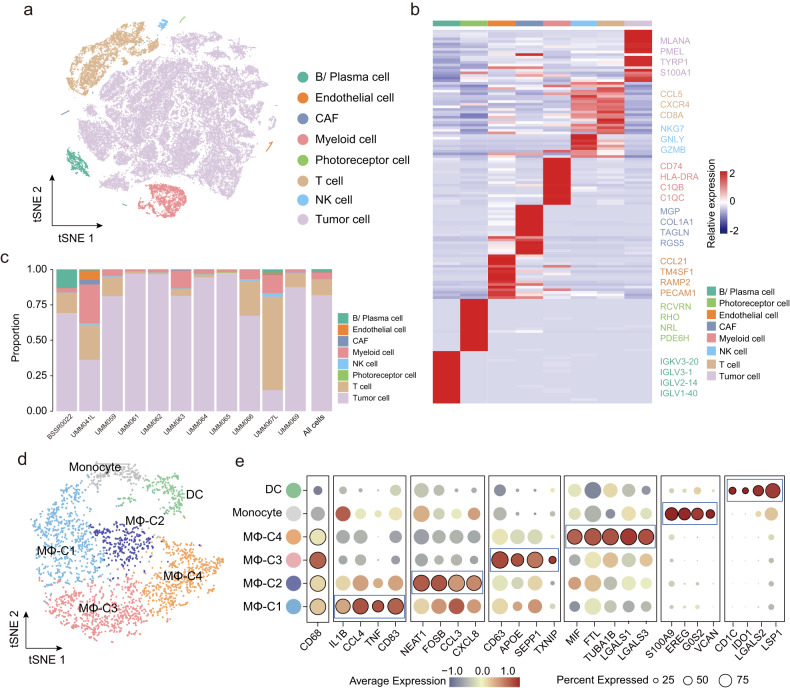
Single-cell transcriptomic analysis revealed four transcriptionally distinct macrophage subsets in UM. **a** t-SNE plot of all single cells color-coded according to their assigned major cell type. **b** Heatmap showing average expression of the top known markers in the indicated cell clusters. **c** The proportion of eight major cell types shown in bar plots in different patients. **d** t-SNE plot displaying six subclusters of 2847 myeloid cells. Each subcluster is color-coded according to cell subsets. **e** Bubble heatmap visualizing marker gene expression for each myeloid subset, where the color and dot size indicate the averaged scaled expression level and percentage of gene expression for each myeloid subset, respectively.

To explore cellular heterogeneity within the TME of UM, we conducted a retrospective analysis of scRNA-seq data for 11 UM patients. Following quality control and batch effect correction, a total of 63,264 qualified cells with a mean of 1555 expressed genes were retained for dimensionality reduction and unsupervised graph-based cell clustering analysis. This analysis identified eight major cell clusters, visualized via t-SNE and manually annotated based on canonical gene markers listed in the Cellmarker database and previously published data^28,40^. These cell clusters include tumor cells (MLANA+, MITF+, and PRAME+), T cells (CCL5+, CXCR4+, CD8A+), NK cells (NKG7+, GNLY+, GZMB+), B/Plasma cells (IGL3-1+, IGLV2-14+, IGLV1-40+), myeloid cells (C1QB+, C1QC+ and CD74+), cancer-associated fibroblasts (CAF, MGP+, RGS5+, COL1A1+), and very small fractions of other cell types: endothelial cells (PECAM1+, RAMP2+ and CCL21+) and photoreceptor cells (RCVRN+) (Fig. 1a–c).

Compared to GEP class 1 tumors and primary tumors, myeloid cells showed relatively higher infiltration abundance in GEP class 2 tumors or metastatic tumors (Supplementary Fig. 1a), indicating the association between myeloid cell infiltration and aggressive tumor behaviors. We further performed SNN-based clustering of 2847 qualified myeloid cells based on 3000 HVGs to dissect the cell composition within an infiltrated myeloid cell compartment and identified six clusters: monocytes, dendritic cells (DCs), and four macrophage subsets (termed MΦ-C1 to MΦ-C4) (Fig. 1d). Monocytes displayed high expression of monocyte-related genes such as S100A9, EREG, G0S2, and VCAN, whereas DCs expressed CD1C, IDO1, LGALS2, and LSP1 (Fig. 1e). For macrophages, MΦ-C1 and MΦ-C2 expressed high levels of proinflammatory mediators (such as IL1B, CCL3, CCL4, FOSB and CD83) and chemokines (CXCL8), and MΦ-C3 highly expressed M2-MΦ markers (such as APOE, SEPP1 and TXNIP). In contrast, MΦ-C4 did not express high levels of the M1/M2 signature genes but instead showed high expression of microtubule-related genes (TUBA1B), ferritin light chain (FTL), and several immunomodulatory molecules (migration inhibitory factor (MIF), Galectin-1 (GAL1) and Galectin-3 (GAL3)). Further enrichment analysis of M1- and M2-MΦ canonical signatures for the four macrophage clusters also indicated that high M1-MΦ signature enrichment scores characterized MΦ-C2 clusters; the MΦ-C3 cluster exhibited high M2-MΦ signature enrichment scores, and MΦ-C1 was characterized by both M1/M2-MΦ signature enrichment scores. Notably, the MΦ-C4 cluster had lower enrichment scores for M1 and M2-MΦ signatures (Supplementary Fig. 1b). Using the average gene expression values of each myeloid subset, we calculated the Spearman’s correlation coefficient for all subsets based on their transcriptional patterns and found a distinct transcriptional profile for the MΦ-C4 cluster that differentiated it from the other three macrophage clusters (Supplementary Fig. 1c).

### Functional characterization of different macrophage subsets in UM

**Fig. 2:**
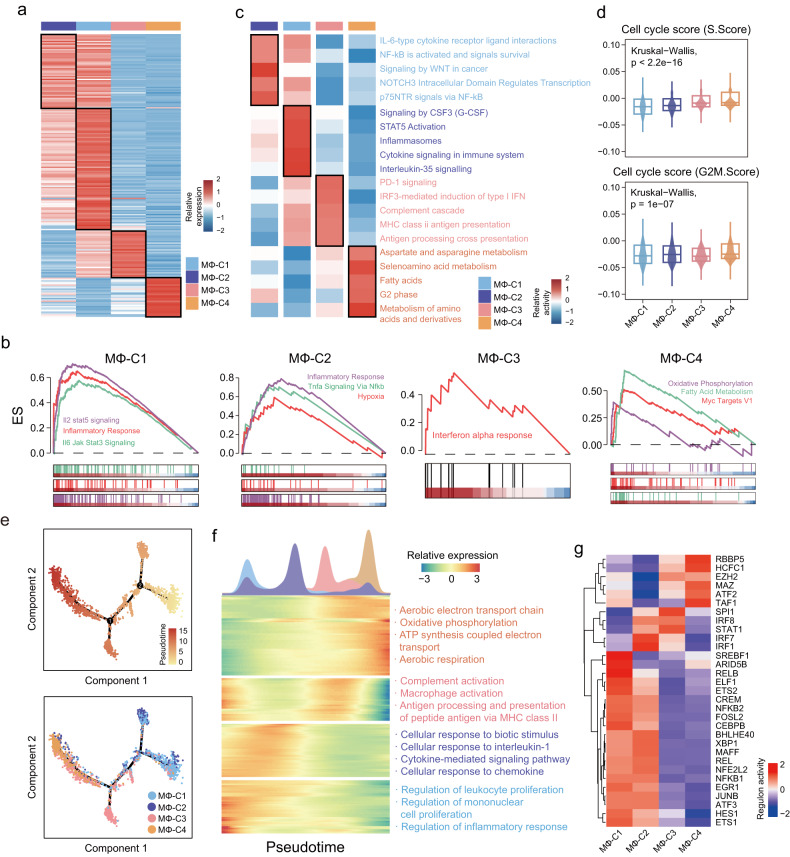
Functional characterization of different macrophage subsets. **a** Heatmap of differentially expressed genes (DEGs) of four macrophage subsets. **b** GSEA enrichment plot for upregulated gene sets in each macrophage subset. **c** Heatmap displaying the activity of statistically enriched Reactome pathways for different macrophage subsets. **d** Violin plots of cell cycle scores for different macrophage subsets. Statistical differences were determined by the Kruskal‒Wallis test. **e** The potential polarization trajectory of different macrophage subsets inferred by Monocle 2. The polarization directions are shown in the top panel, and the distribution of each macrophage subset during the developmental trajectory is shown in the bottom panel. **f** Differentially expressed genes along the pseudotime were clustered hierarchically into four profiles. Representative gene functions and pathways are shown. **g** Heatmap displaying the regulon activity of statistically enriched transcription factors for different macrophage subsets estimated by SCENIC.

To investigate the functional heterogeneity of macrophage subsets, we performed single-cell differential expression analysis between one macrophage subset and three other macrophage subsets to identify significantly upregulated genes in specific subsets (Fig. 2a and Supplementary Table 2). Functional enrichment analysis for cancer hallmark pathways revealed the upregulated genes of MΦ-C1 to be enriched in IL-6/JAK/STAT3 signaling, IL-2/STAT5 signaling, and inflammatory response, TNF-α signaling via NF-κB, hypoxia and inflammatory response in MΦ-C2, and interferon alpha response in MΦ-C3. Oxidative phosphorylation, fatty acid metabolism, and MYC Targets V1 were enriched in MΦ-C4 (Fig. 2b). Additionally, gene set variation analysis was applied to calculate the activity of biological functions based on the Reactome database. The results showed that ‘IL-6-type cytokine receptor‒ligand interactions’, ‘NF-kB is activated and signals survival’, ‘signaling by WNT in cancer’, ‘NOTCH3 Intracellular Domain Regulates Transcription’, and ‘p75NTR signals via NF-kB’ were enriched in MΦ-C2, and MΦ-C3 was enriched in ‘PD-1 signaling’, ‘IRF3-mediated induction of type I IFN’, ‘Complement cascade’ and ‘MHC class II antigen presentation’. MΦ-C1 not only shared similar pathways with MΦ-C2 and MΦ-C3 but was also enriched in interleukin-35 signaling and cytokine signaling in the immune system. In contrast, metabolism-related pathways and the G2 phase were exclusively enriched in MΦ-C4, suggesting potential metabolic and proliferative properties unique to this subset (Fig. 2c). Therefore, we utilized Seurat to computationally define cell cycle phase scores for each macrophage subset based on expression levels of S and G2/M phase markers. As shown in Fig. 2d, MΦ-C4 had higher S and G2/M phase scores than the other three macrophage subsets (Fig. 2d).

Moreover, the heterogeneity of innate immune functions was investigated among these four macrophage subsets, with the results showing the lowest enrichment of innate immune gene signatures for MΦ-C4, indicating potential dysfunction in innate immunity (Supplementary Fig. 2a). Further examination of the Toll-like receptor (TLR) family demonstrated reduced expression levels in MΦ-C4 compared to the other three macrophage subsets (Supplementary Fig. 2b). TLRs are pattern recognition receptors critical in initiating the innate immune response in macrophages. This suggests that TLR deficiency may partly contribute to dysfunctional innate immunity in MΦ-C4.

Trajectory analysis was subsequently performed using Monocle to investigate the polarization trajectories of macrophages in UM. This trajectory analysis indicated a branched structure, starting with MΦ-C1 and MΦ-C2 and bifurcating into multiple macrophage polarization states (Fig. 2e). As illustrated in Fig. 2e, MΦ-C1, MΦ-C2, and MΦ-C3 are more redundant in the early and middle stages of the trajectory, respectively, whereas MΦ-C4 is primarily at the terminal branch. For validation, we used another trajectory reconstruction computational tool, cytoTRACE, to reproduce the differentiation trajectory of macrophages, with MΦ-C1 and MΦ-C2 having the highest developmental potential and MΦ-C4 in the final differentiated state (shown as low developmental potential, Supplementary Fig. 2c, d). Transcriptional and functional changes were explored across pseudotime. Enrichment analyses of DE genes across pseudotime indicated the inflammatory response, cytokine-mediated signaling pathway, and complement activation to be activated in the early and middle stages; however, they were uniformly downregulated in the late stage of polarization (Fig. 2f). In contrast, metabolic pathways and ATP biosynthetic processes involved in cellular energetics showed high enrichment in the late stage of polarization (Fig. 2f).

To identify key transcription factors (TFs) determining the state of each macrophage subset, the SCENIC method was applied to determine the correlation between TFs and different transcriptional programs among macrophage subsets and identify multiple TF activity patterns (Fig. 2g). Inflammation-related TFs (*NFKB1*, *NFKB2*, *JUNB*, and *FOSB*) were activated in MΦ-C1 and MΦ-C2, whereas classical interferon regulatory TFs (*IRF* and *STAT*) were identified in MΦ-C3. A total of six TFs (*EZH2*, *RBBP5*, *ATF2*, *TAF1*, *HCFC1*, and *MAZ*) were highly elevated in MΦ-C4, which might mediate the phenotype and characteristics of MΦ-C4.

### Single-cell association of transcriptionally distinct macrophage subsets with UM aggressive behaviors

**Fig. 3:**
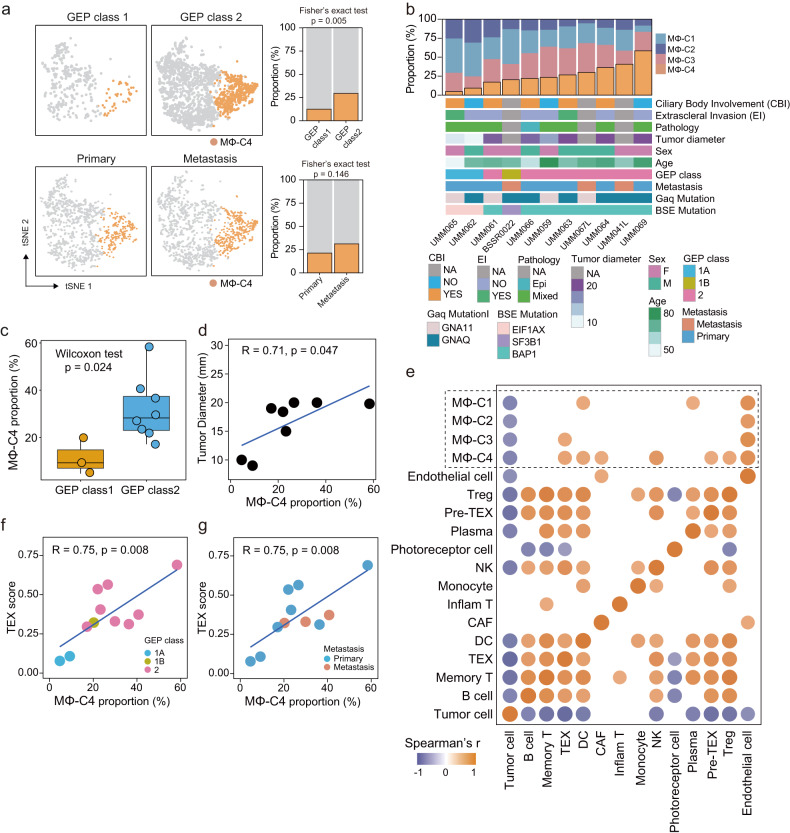
Single-cell association of transcriptionally distinct macrophage subsets with UM aggressive behaviors. **a** Comparison of MΦ-C4 proportions between GEP class 1 and GEP class 2 tumors and primary and metastatic tumors from single-cell data. Statistical differences were determined by Fisher’s exact test. **b** The MΦ-C4 proportions and landscape of clinical characteristics among each patient. **c** Boxplots showing differences in MΦ-C4 proportions between GEP class 1 and GEP class 2 tumors. Statistical differences were determined by the Wilcoxon rank-sum test. **d** Scatter plots showing the correlation of the proportion of MΦ-C4 and tumor diameter. Correlation was evaluated by the Spearman correlation coefficient. **e** Heatmap showing the Spearman correlation coefficients between proportions of different cell subsets. *P* < 0.05 was considered the cutoff value. **f** Scatter plots showing the correlation of the proportion of MΦ-C4 and T-cell exhaustion (TEX) scores. Correlation was evaluated by the Spearman correlation coefficient, and the dot color represents the GEP classification. **g** Scatter plots showing the correlation of the proportion of MΦ-C4 and T-cell exhaustion (TEX) scores. Correlation was evaluated by the Spearman correlation coefficient, and the dot color represents the primary tumor or metastasis.

To investigate the clinical relevance of transcriptionally distinct macrophage subsets, we examined differences in infiltration abundance at both the single-cell and patient levels. Relative proportions of different macrophage subsets in GEP class 1 and GEP class 2 tumors and primary and metastatic tumors from single-cell data were determined. The relative proportion of MΦ-C4 was higher in GEP class 2 tumors (29.39%) and metastatic tumors (32.84%) than in GEP class 1 tumors (12.47%) and primary tumors (22.98%; Fig. 3a). Figure 3b shows that MΦ-C4 was commonly present in each UM sample, though the level differed between samples, ranging from 4.7% to 58.3%. Furthermore, when analyzing the correlation between infiltration abundance and clinical features, we found that the infiltrating density of MΦ-C4 was higher in GEP class 2 tumors (*P* = 0.024) than in GEP class 1 tumors (Fig. 3c, Supplementary Fig. 3a) and MΦ-C4 infiltrated more in metastatic tumors (Supplementary Fig. 3b). In addition, the tumor diameter correlated significantly positively with the infiltrating density of MΦ-C4 compared to other macrophage subsets (*r* = 0.71 and *P* = 0.047; Fig. 3d, Supplementary Fig. 3c). These results suggest that increased infiltration of MΦ-C4 is related to aggressive behaviors.

To investigate the interplay between macrophage subsets and other cell components, we further resolved T cells into eight subsets based on established markers: memory T cells, regulatory T cells (Tregs), inflammation-related T cells expressing TNF and IFNG (Inflam T), and preexhausted (Pre-TEX) and exhausted T cells (TEX) expressing moderately and highly exhausted markers (Supplementary Fig. 4a, b). B cells and plasma cells were also differentiated (Supplementary Fig. 4c, d). Spearman’s rank correlation coefficients were computed for each subset (Fig. 3e). In contrast to other macrophage subsets, MΦ-C4 correlated highly with the tumor-derived population of cancer-associated fibroblasts (CAFs) and immunosuppressive T lymphocyte subsets, including Tregs, preexhausted T cells (Pre-TEX), and exhausted T cells (TEX) (Fig. 3e). A significant positive association between MΦ-C4 and T-cell exhaustion (TEX) was also observed (*r* = 0.75 and *P* = 0.008, Fig. 3f and g).

### High infiltration of MΦ-C4 correlates with poor prognosis

**Fig. 4:**
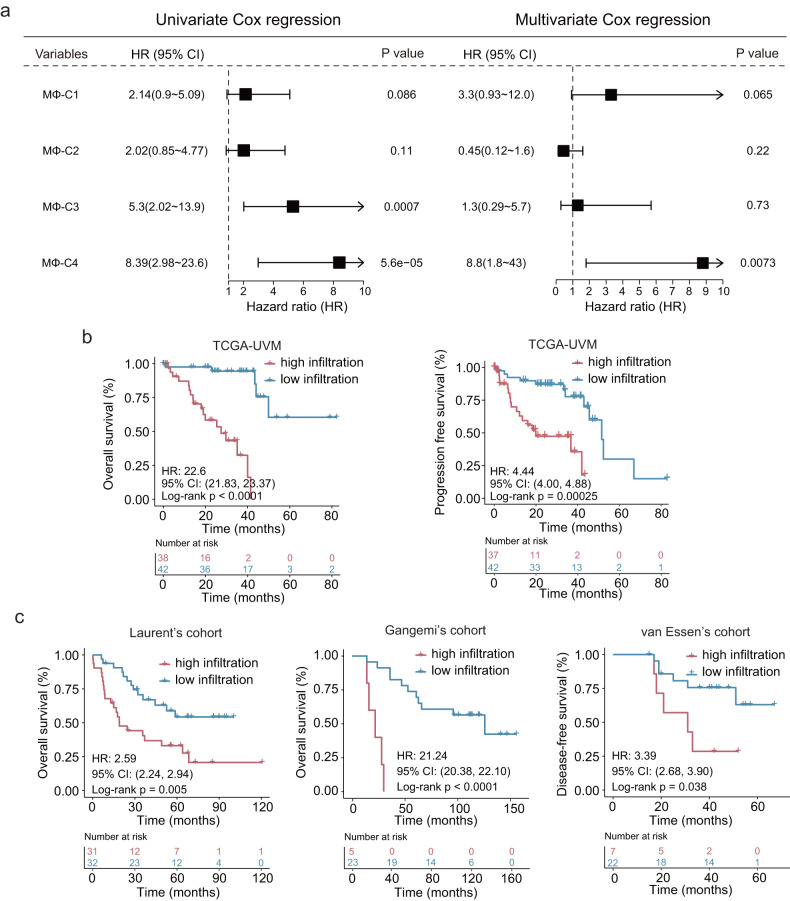
High infiltration of MΦ-C4 correlates with poor prognosis. **a** Forest plots showing hazard ratios and confidence intervals (horizontal ranges) derived from univariate and multivariable Cox regression analyses for overall survival for each macrophage subset in TCGA cohorts. **b** Kaplan–Meier survival curves for overall survival (left panel) and progression-free survival (right panel) for patients with high and low MΦ-C4 infiltration in the TCGA cohort. **c** Kaplan–Meier survival curves for overall survival and disease-free survival for patients with high and low MΦ-C4 infiltration in three GEO multicenter cohorts.

Considering the heterogeneity of MΦ-C4 in UM patients and its association with aggressive behaviors observed at the single-cell level, we investigated its clinical implications in a cohort of 80 UM patients from TCGA. Using ssGSEA and scRNA-seq-defined overexpressed markers, we estimated the infiltration abundance of different macrophage subsets. Multivariate Cox analysis revealed that only MΦ-C4 remained significantly associated with overall survival (OS) (HR, 8.8; 95% CI, 1.8–43; *p* = 0.0073), suggesting that MΦ-C4 is an independent prognostic risk factor for UM patients (Fig. 4a). Survival analysis showed that patients with high MΦ-C4 infiltration had significantly shorter OS (HR, 22.6; 95% CI, 21.83–23.37; log-rank *P* < 0.0001) and progression-free survival (HR, 4.44; 95% CI, 4.00–4.88; log-rank *P* = 0.00025) than those with low MΦ-C4 infiltration (Fig. 4b). Additionally, the findings were consistent across three external GEO cohorts, whereby patients with high MΦ-C4 density showed poor survival compared to those with low MΦ-C4 density (HR, 2.59; 95% CI, 2.24–2.94; log-rank *P* = 0.005 for Laurent’s cohort; HR, 21.24; 95% CI, 20.38–22.10; log-rank *P* < 0.0001 for Gangemi’s cohort; and HR, 3.39; 95%, CI 2.68–3.90; log-rank *P* = 0.038 for van Essen’s cohort; Fig. 4c). After adjusting for clinical variables, including locational information, age, tumor basal diameter, sex, histopathology, and tumor stage, multivariate Cox regression analysis revealed that high infiltration of MΦ-C4 remained significantly associated with poor survival in four UM cohorts (TCGA: HR 57, 95% CI 9.9–320, *P* < 0.001; Laurent’s: HR 3.7, 95% CI 1.7–8.4, *P* = 0.0015; Gangemi’s: HR 18, 95% CI 3.2–100, *P* = 0.0011; van Essen’s: HR 4, 95% CI 0.89–18, *P* = 0.072) (Supplementary Fig. 5). These findings suggest that MΦ-C4 infiltration may be an independent and robust prognostic factor for UM patients.

### Potential MΦ-C4-based UM subtyping

**Fig. 5:**
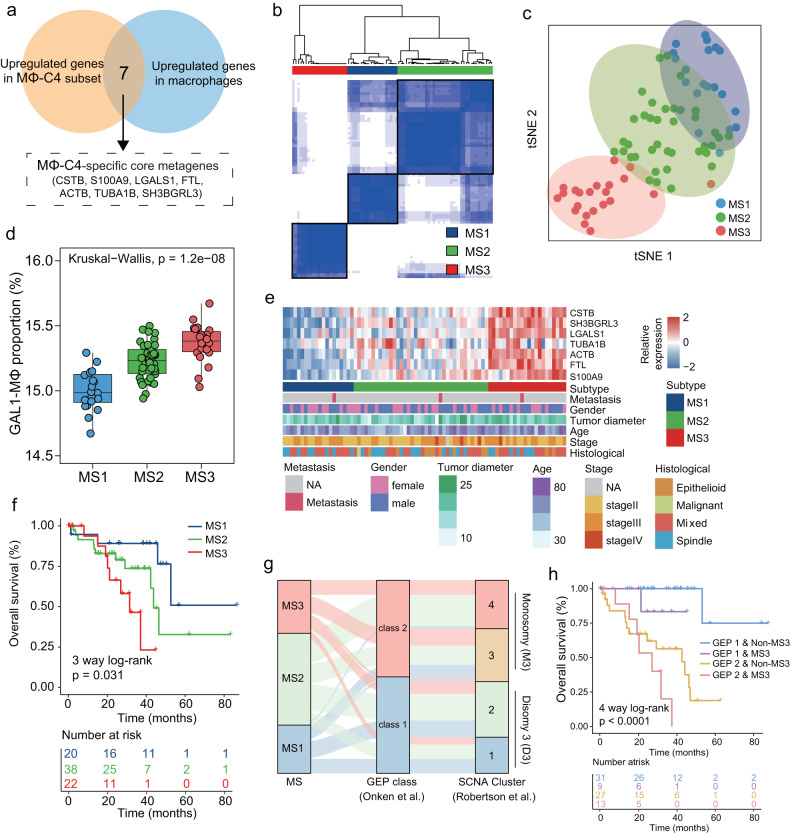
Potential MΦ-C4-based UM subtyping. **a** Venn diagram illustrating MΦ-C4 core metagenes. **b** Consensus clustering for TCGA-UVM based on seven core metagenes. **c** t-SNE analysis of the three subtypes. **d** Box plots showing the different estimated MΦ-C4 proportions in the different subtypes. Statistical differences were determined by the Kruskal‒Wallis test. **e** The relative expression level of MΦ-C4 core metagenes and landscape of clinical characteristics among different subtypes. **f** Kaplan–Meier survival curves for overall survival for three subtypes in the TCGA cohort. **g** Alluvial diagram showing the association between MΦ-C4-defined subtypes and previously defined molecular subtypes. **h** Kaplan–Meier survival curves for overall survival for different molecular subtypes.

Given the heterogeneity of UM, we sought to determine whether MΦ-C4 within the TME can be used to define UM subtypes. We conducted differential gene expression analysis to identify MΦ-C4-specific core metagenes by comparing the gene expression profiles of macrophages with those of other cell types and of MΦ-C4 with those of other macrophage subsets. Finally, seven genes (*CSTB*, *S100A9*, *LGALS1*, *FTL*, *ACTB*, *TUBA1B*, *SH3BGRL3*) were identified as MΦ-C4-specific core metagenes (Fig. 5a). Then, we performed consensus clustering analysis for UM patients in the TCGA cohort based on MΦ-C4-specific core metagenes and identified three patient clusters, designated MS1, MS2 and MS3 (Fig. 5b), which were reproducible through clustering of spectral patterns using t-SNE dimensionality reduction (Fig. 5c). All seven MΦ-C4-specific core metagenes were upregulated in MS3, and the abundance of infiltrating MΦ-C4 was significantly higher in MS3 than in MS1 and MS2 (Fig. 5d, e). Survival analysis revealed that MS1 and MS2 were associated with improved prognosis but that MS3 had the poorest survival probability (log-rank *P* = 0.031; Fig. 5f). Moreover, the distribution of subtypes across different clinicopathological characteristics indicated nonuniform clustering of patients (Fig. 5e). We further assessed the interplay of MΦ-C4-based subtyping with gene expression profiling (GEP)-based or previously defined somatic copy number alteration (SCNA)-based classification (Fig. 5g). As depicted in Fig. 5h, MΦ-C4-based subtyping can effectively stratify UM patients in different GEP classes or SCNA subsets into different risk groups with distinct survival (four-way log-rank *P* < 0.0001) (Fig. 5h). For example, GEP class 2 tumors in non-MS3 were associated with better OS than GEP class 2 tumors in MS3. These results suggest that molecular subtyping based on MΦ-C4 provides additional prognostic value beyond traditional staging and known molecular subtyping.

### Development of an MΦ-C4-derived subtyping system for UM molecular diagnosis and prognosis prediction

**Fig. 6:**
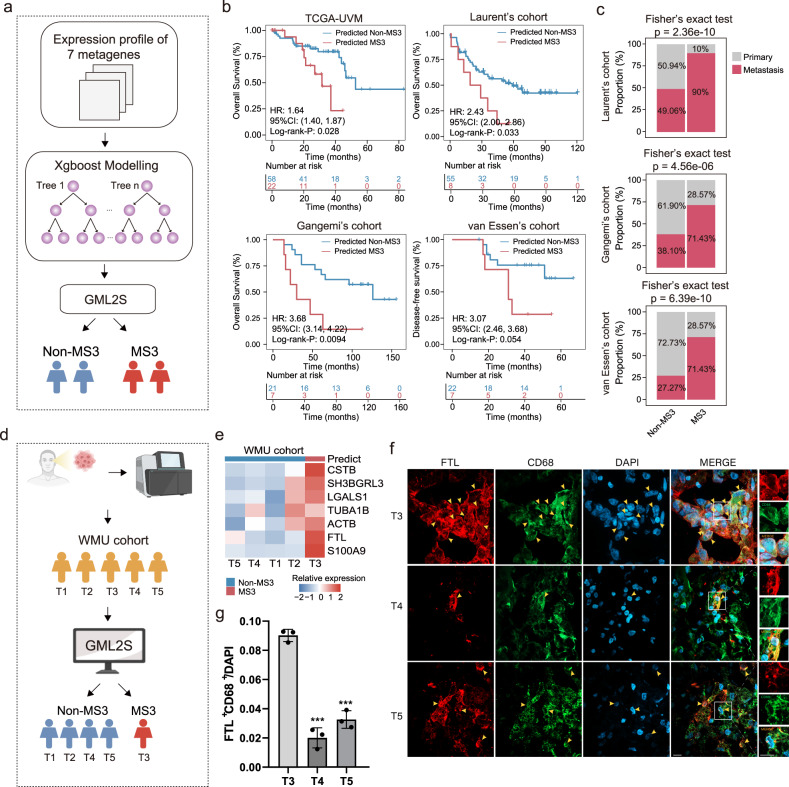
Development of an MΦ-C4-derived subtyping system for UM molecular diagnosis and prognosis prediction. **a** Schematic representation of MΦ-C4-based molecular subtyping. **b** Kaplan–Meier survival curves for overall survival for patients with MS3 and non-MS3 subtypes in the TCGA cohort and three GEO multicenter cohorts. **c** Bar plot showing the percentage of primary and metastatic patients between predicted MS3 and non-MS3 subtypes in three GEO multicenter cohorts. Statistical differences were determined by Fisher’s exact test. **d** Schematic representation of applying the MΦ-C4-derived subtyping system in the WMU cohort. **e** Heatmap showing the relative expression level of 7 metagenes among predicted MS3 and non-MS3 subtypes in the WMU cohort. **f** Immunofluorescence imaging of three representative cases with dual staining of FTL (red) and CD68 (green), alongside DAPI (blue), in individual and merged channels are shown. The scale bar represents 25 μm. **g** Quantification of CD68+ and FTL+ cells. Values were normalized to the total number of DAPI+ cells. Error bars represent the mean ± SEM. Statistical analysis was performed using an unpaired two-tailed Student’s *t*-test, (*) = *p* < 0.05.

To accelerate the potential clinical applications of MΦ-C4-based molecular subtyping, we utilized a gradient-boosting machine-learning framework to establish a subtyping system (termed GML2S) that classifies UM subtypes and predicts prognosis based on MΦ-C4-specific core metagenes (Fig. 6a). We trained GML2S using the TCGA UM cohort and validated it using external UM cohorts. The MS3 subtype identified using GML2S showed significantly shorter survival times than those with predicted non-MS3 subtypes (log-rank *P* = 0.028 for TCGA cohort; log-rank *P* = 0.033 for Laurent’s cohort; *P* = 0.0094 for Gangemi’s cohort and *P* = 0.054 for van Essen’s cohort; Fig. 6b). Moreover, GML2S was highly informative for identifying patients with metastasis (Fig. 6c).

GML2S was further applied to five UM samples from the in-house WMU cohort for subtype classification. Among these samples, one patient was predicted to have the MS3 subtype; the remaining four patients were predicted to have the non-MS3 subtype by GML2S (Fig. 6d). As indicated in Fig. 6e, the patients with the predicted MS3 subtype exhibited significantly higher expression levels of MΦ-C4-specific core metagenes than the non-MS3 patients (Fig. 6e). Immunofluorescence microscopy was performed on tissues from three representative cases from the WMU cohort to confirm increased infiltration of MΦ-C4 in the predicted MS3 case compared with the remaining two non-MS3 cases (Fig. 6f, g). These results highlight the potential of GML2S in UM molecular diagnosis and subtype classification.

## Discussion

The immune microenvironment is a critical regulator of tumor initiation, progression, and therapeutic response due to its complexity and diversity^41–44^. Macrophages, among the most common infiltrating immune cells in the tumor immune microenvironment, play a dual role in promoting and inhibiting tumor growth and progression^45,46^. With the advent of scRNA-seq, there is increasing evidence of the remarkable plasticity and broad spectrum of macrophages, leading to the identification of diverse macrophage subsets in multiple cancer types^24^. However, the heterogeneity of macrophages and their clinical significance in UM require further elucidation.

In the present study, our single-cell RNA-seq analysis provides a comprehensive understanding of the cellular heterogeneity of macrophages in UM, revealing distinct transcriptional patterns and functions of four heterogeneous macrophage populations. The findings also indicate that the conventional M1/2 classification may not fully characterize the macrophage subsets involved in UM, as macrophages exhibit complex multifunctional phenotypes. Moreover, a unique macrophage population (MΦ-C4) with a distinct transcriptomic signature and characteristics was identified, as characterized by proliferation signaling and metabolic capacity. Dramatic alterations in cell metabolic profiles have been used to characterize the phenotype and function of macrophages^47^.

The results of the present study demonstrate that core regulons in MΦ-C4 inferred from SCENIC analysis are involved in cell proliferation and mitochondrial biogenesis, which may play crucial roles in coordinating the phenotype of MΦ-C4. HCFC1 controls the cell cycle from G1 to S phase in multiple ways, and MYC can interact with HCFC1 to drive ribosome biogenesis and mitochondrial programs^48,49^. ATF2 was proven to directly regulate cyclin B1, cyclin D1, etc., and was associated with poor prognosis in a study of ccRCC^50^. As a potential E2F target, EZH2 is an important marker for cell proliferation^51^. In addition to proliferation and metabolism signaling, MΦ-C4 increases the expression of multiple immunomodulatory molecules, including GAL1, GAL3, and MIF. GAL1 accelerates tumor growth and immune escape by inducing cytotoxic T-cell apoptosis and may be a promising immune checkpoint^52,53^. GAL3 is a multifunctional immunosuppressive ligand and recruits T-cell subsets highly expressing immune checkpoint molecules into the tumor microenvironment, and MΦ-C4-enriched UMs tended to have high infiltration of exhausted T cells and Tregs^54^. MIF is reported as an essential effector molecule for inhibiting the cytolytic activity of NK cells^55^ and highlights the role of MIF in the upregulation of multiple oncogenic pathways, leading to tumor malignancies and progression^56–58^. In addition, we found that the MΦ-C4 identified in our study resembles a subset of C1QC+TAMs in Zhang’s study (Supplementary Fig. 4e). These results indicate the protumorigenic effects of MΦ-C4 in UM, but its potential clinical significance requires further investigation.

Notably, the association between MΦ-C4 infiltration, aggressive behaviors, and poor survival outcomes was observed in both scRNA-seq and TCGA bulk RNA-seq cohorts, and among the macrophage subsets, MΦ-C4 was observed to have the worst prognosis in UM. These results were validated using multicenter microarray cohorts. The clinical effects of MΦ-C4 infiltration not only depend on the technology used to measure gene expression (scRNA-seq, bulk RNA-seq, or microarray) but are also independent of important clinical variables. These data indicate that MΦ-C4 may have potential as a therapeutic target.

Cancer subtyping allows for understanding tumor heterogeneity and improving risk stratification and clinical decisions^59,60^. Several molecular subtypes have been established for UM, but these are limited to genetic, genomic, transcriptomic, and methylomic subtypes^2,9,11,61^. Following the observed clinical significance of MΦ-C4 infiltration, by incorporating the transcriptional signatures of MΦ-C4 and machine learning, this subtyping scheme can potentially provide additional information on the immune microenvironment of UM tumors and aid in more informed clinical decisions. The validation of this subtyping scheme in multiple public UM cohorts and the in-house cohort of the present study, as well as its confirmation through immunofluorescent imaging, supports its potential clinical utility. However, further studies are necessary to fully understand the implications of this subtyping scheme and its ability to improve risk stratification and clinical outcomes for UM patients.

In conclusion, our study utilized single-cell transcriptome data to characterize the transcriptional heterogeneity of macrophages within the UM TME and demonstrated the clinical relevance of these macrophage subsets for assessing disease aggressiveness and prognosis. These findings deepen our knowledge of cellular heterogeneity in UM and emphasize the potential therapeutic benefits of targeting macrophages in UM treatment.

### Supplementary information

Supplementary Materials

Supplementary Table S1

Supplementary Table S2

## Data Availability

The raw RNA-seq data generated during this study have been deposited in the Gene Expression Omnibus (GEO) database (https://www.ncbi.nlm.nih.gov/geo/, GSE211763). All public single-cell RNA sequencing data and bulk microarray data are available from the GEO database under accession numbers GSE139829, GSE22138, GSE27831, and GSE84976. Public bulk RNA-seq data were obtained from UCSC Xena (GDC TCGA-UVM cohort) (https://xenabrowser.net/datapages/).

## Declarations

This study was performed according to the Declaration of Helsinki and approved by the human ethics committee at the Eye Hospital of Wenzhou Medical University (2022-043-K-28-02). All patients consented to the study and signed a written form. All data were anonymously analyzed. The study was performed in accordance with the regulations and guidelines established by this committee.
