# Supplementary material for: Single-cell characterization of macrophages in uveal melanoma uncovers transcriptionally heterogeneous subsets conferring poor prognosis and aggressive behavior
Source: Exp Mol Med. 2023 Nov 1;55(11):2433–44. doi: 10.1038/s12276-023-01115-9 (PMC10689813; doi:10.1038/s12276-023-01115-9)
Supplement: Supplementary file 1 — Supplementary Materials [file 12276_2023_1115_MOESM1_ESM.pdf]

Supplementary Materials

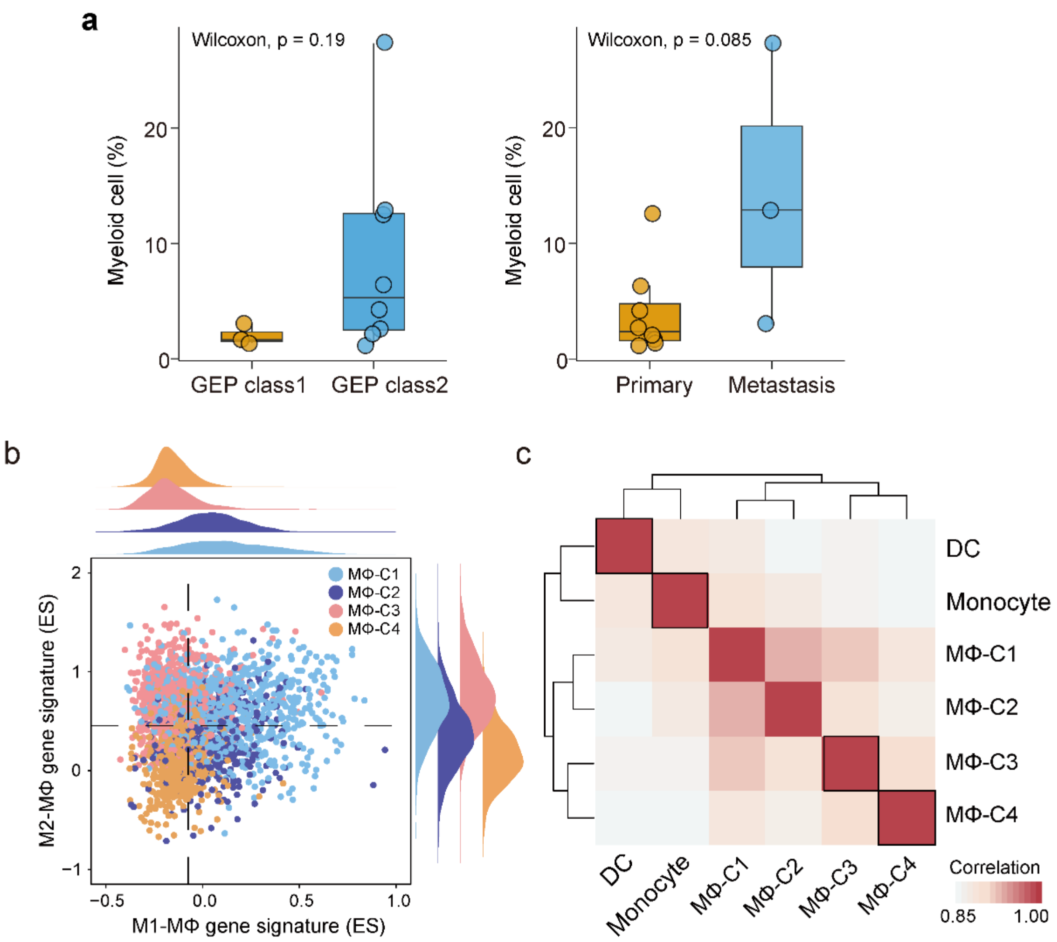

Supplementary Fig. 1. (a)Boxplots showing the proportion differences of myeloid cells between GEP class 1 and GEP class 2 tumors, and between primary and metastasis tumors. Statistical differences were determined by the Wilcoxon rank-sum test. (b) Scatter plot showing M1- and M2-macrophages gene signature enrichment scores for four macrophage subsets. (c) Clustering heatmap visualizing spearman correlation among different myeloid subsets.

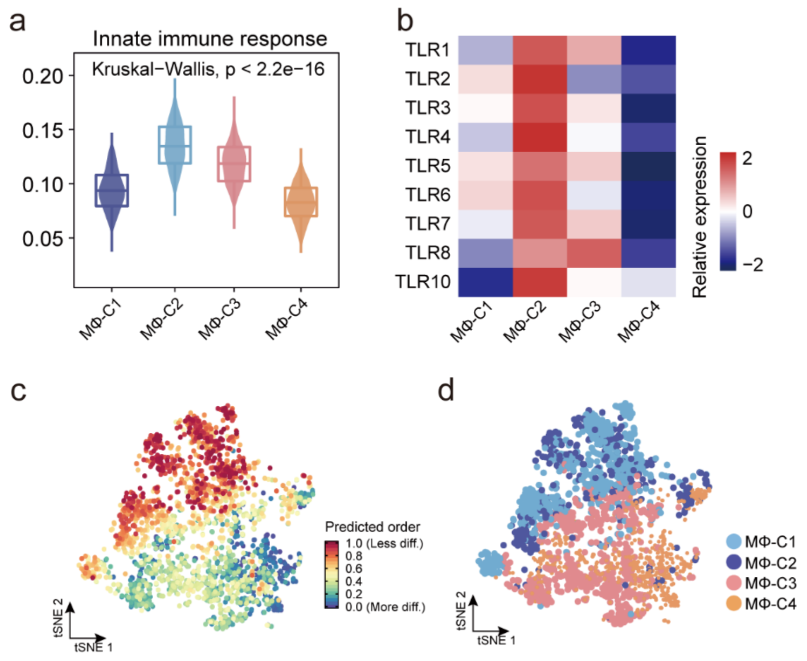

Supplementary Fig. 2. (a) Violin plots of the innate immune score for different macrophage subsets. (b) Heatmap showing the expression of toll-like receptor genes for different macrophage subsets. (c) t-SNE plot displaying the differentiation potential of macrophages predicted by CytoTRACE. Each cell is color-coded according to the potential differentiation score. (d) t-SNE plot displaying the distribution of macrophages predicted by CytoTRACE. Each cell is color-coded according to cell type.

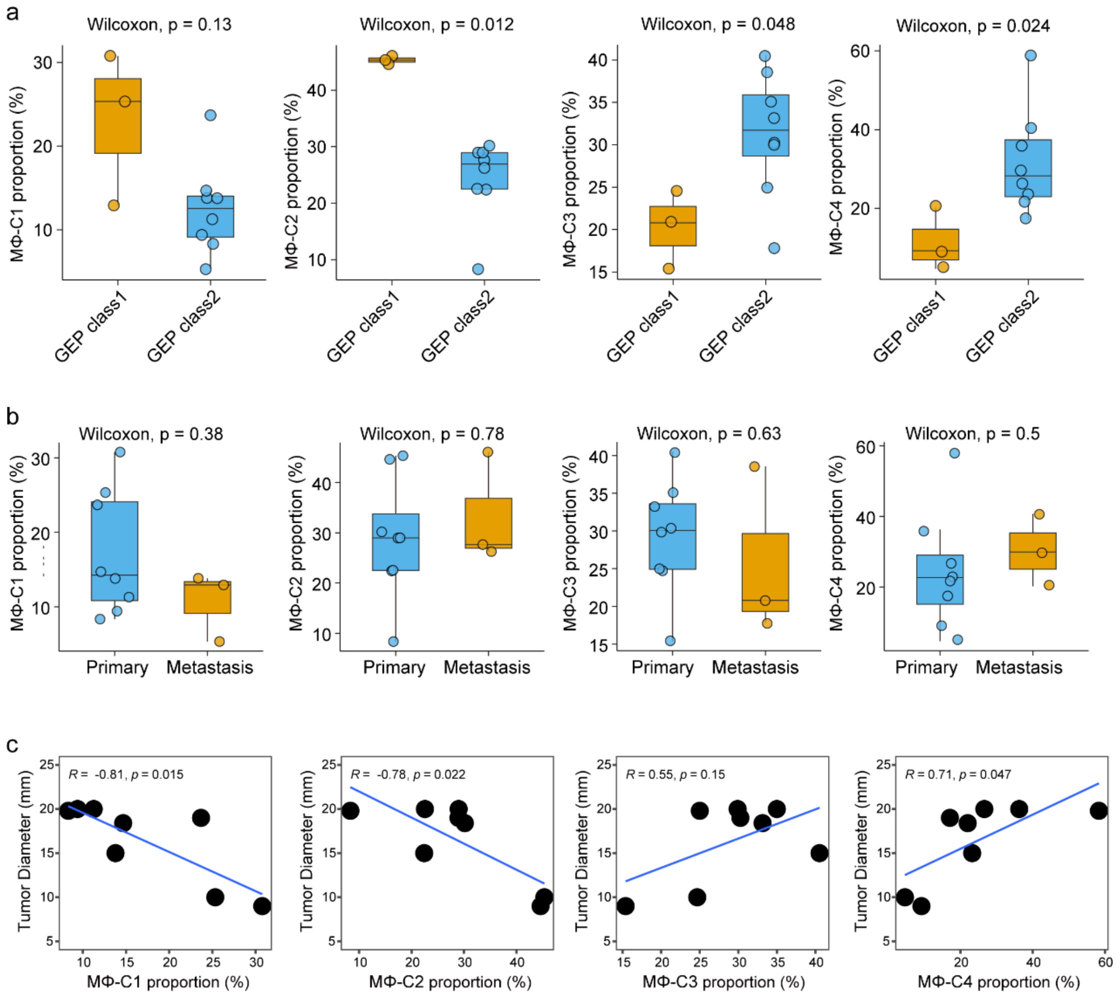

Supplementary Fig. 3. (a) Boxplots showing the proportion differences of different macrophage subsets between GEP class 1 and GEP class 2 tumors. Statistical differences were determined by the Wilcoxon rank-sum test. (b) Boxplots showing the proportion differences of different macrophage subsets between primary and metastasis tumors. Statistical differences were determined by the Wilcoxon rank-sum test. (c) Scatter plots showing the correlation of the proportion of different macrophage subsets and tumor diameter. Correlation is evaluated by the Spearman correlation coefficient.

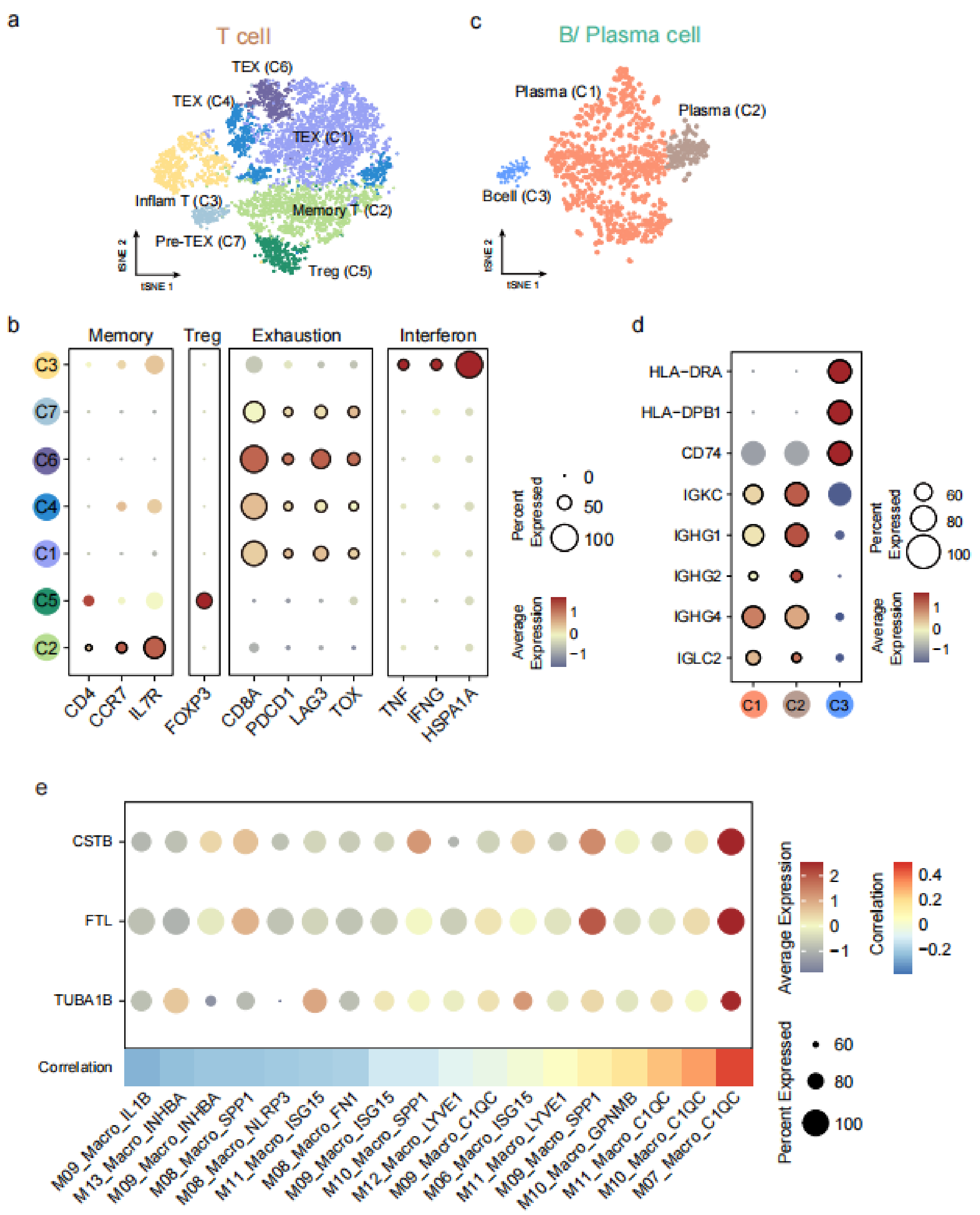

Supplementary Fig. 4. (a) t-SNE plot displaying eight subclusters on 6793 T cells. Each subcluster is color-coded according to cell subsets. (b) Bubble heatmap visualizing marker gene expression for each T cell subset, where the color and dot size encodes the averaged scaled expression level and percentage of gene expression for each subset, respectively. (c) t-SNE plot displaying eight subclusters on 1160 B/ Plasma cells. Each subcluster is color-coded according to cell subsets. (d) Bubble heatmap

visualizing marker gene expression for each B/ Plasma subset, where the color and dot size encodes the averaged scaled expression level and percentage of gene expression for each subset, respectively. (e) Bubble heatmap visualizing marker gene expression of MΦ-C4 for each macrophage subset in Zhang's study, where the color and dot size encodes the averaged scaled expression level and percentage of gene expression for each subset, respectively. The annotation color represented the Spearman correlation coefficient between macrophage subsets in Zhang's cohort and MΦ-C4.

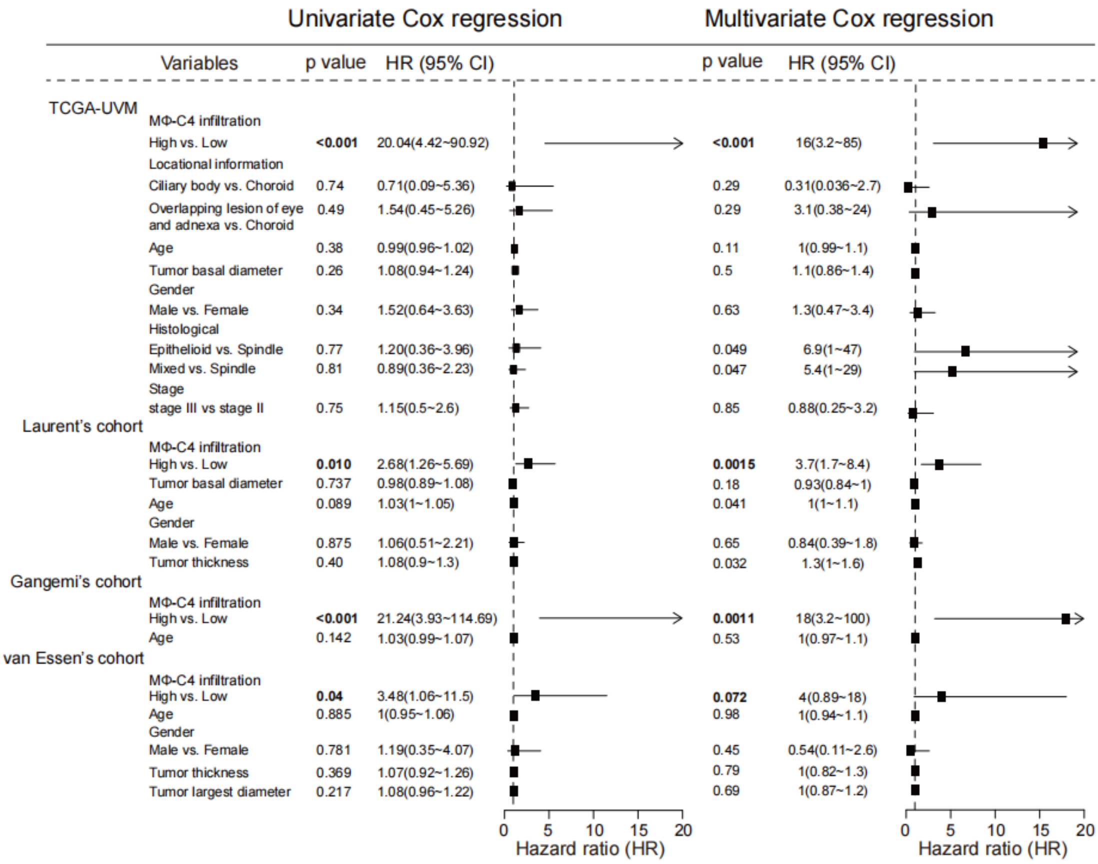

Supplementary Fig. 5. Forest plots showing hazard ratio and confidence intervals (horizontal ranges) derived from Cox regression survival analyses for overall survival in multivariable analyses adjusted for locational information, age, tumor basal diameter, gender, histopathology and tumor stage in TCGA and three GEO multicenter cohorts.

Supplementary Table 1. M1- and M2-MΦ signature genes used for calculating M1- and M2-MΦ signature enrichment scores.

Supplementary Table 2. List of macrophage subset-specific up-regulated genes.
